# India’s response to adolescent mental health: a policy review and stakeholder analysis

**DOI:** 10.1007/s00127-018-1647-2

**Published:** 2019-01-03

**Authors:** Kallol Roy, Sachin Shinde, Bidyut K. Sarkar, Kanika Malik, Rachana Parikh, Vikram Patel

**Affiliations:** 1 Sangath, Porvorim, Goa India; 2 Vrije University, Amsterdam, The Netherlands; 3 Department of Global Health and Social Medicine, 641 Huntington Avenue, Boston, MA 02115 USA

**Keywords:** Adolescent, Mental health, Policy, Review, India

## Abstract

**Purpose:**

Mental health problems and suicide are the leading cause of mortality in young people globally. India is home to the largest number of adolescents in the world. This study was undertaken to assess the policy environment for addressing adolescent mental health in India.

**Methods:**

We conducted a review of 6 policies and programs and 11 in-depth interviews with key stakeholders. The findings were analyzed using the policy triangle analysis framework (i.e., context, content, actors and process).

**Results:**

There is no conformity of the age ranges addressed by these documents nor are vulnerable groups explicitly recognized. Stress, anxiety and depression were commonly identified as mental health concerns and diverse platforms such as community, family, school, digital and health facility were recommended to deliver preventive and treatment interventions. Some interventions specifically targeted some social determinants (like safe and supportive schools) but many others (like social norms) were not addressed. Preventive interventions were recommended for delivery through peers and other non-specialist providers while treatment interventions were recommended for delivery in healthcare facilities by specialist health professionals. There was very little engagement of young people in the development of these policies or in their implementation, except for peer educators mentioned in one policy. Stakeholders identified several major challenges in implementing these policies, notably the lack of inter-sectoral coordination and fragmentation of governance; budgetary constraints; and scanty human resources.

**Conclusions:**

Although there are now several policy instruments testifying to a comprehensive approach on adolescent mental health, there are gaps in the extent of engagement of young people and how these will be operationalized that may limit their impact on addressing the burden of mental health problems in young people in India.

## Introduction

Mental health and substance use problems are the leading cause of years lived with disability (YLD) among adolescents and young people (10–23 years) accounting for 22.9% of the total global YLDs [1]. These problems have significant adverse impacts on individual, family and society and are frequently associated with poor academic, occupational, and psychosocial functioning, and contribute to premature mortality through their association with suicide and accident related mortality, both the leading causes of death in this age group [1]. Further, more than half of the burden of mental disorders in adulthood has its onset in adolescence. However, evidence shows a lack of comprehensive policy response to the mental health needs of adolescents in both low- and high-income countries [2]. The recent Lancet Commission on adolescent health and well-being [3], suggested that the focus of health policy needs to expand from infectious diseases to non-communicable diseases, including mental health and substance use.

India is home to the largest number of adolescents in the world, comprising about a fifth of its population (243 million) [4]. A meta-analysis reported that 6.5% of the community samples and 23.3% of school samples experienced significant mental health morbidity [5]. Suicide is the leading cause of death in older adolescents [4]. There has historically been little explicit attention to adolescent mental health in India but, in the past decade, both mental health and adolescent health have received increasing attention in policy and programs. Convergence of policy attention to these focus areas and successful implementation of policies, promise to reduce the burden of mental disorders not just in adolescents but would also make significant contributions to the global burden of mental health disorders. The goal of this paper is to map the policy and program environment for adolescent mental health in India, with the specific objectives of addressing the following research questions:

1. How are adolescent mental health issues addressed in policies and programs?
2. What are the strengths and gaps of these policies and programs with regard to their context, their content, the process of their development and the extent of their implementation?
3. What are the recommendations for impactful implementation of these policies and programs?

## Methods

We conducted two concurrent studies: a review of key policies and programs documents, and semi-structured interviews with key informants.

### Policy and program review

In our study, policies refer to an organized set of values, principles and objectives for improving health and reducing its burden in a given population. It further defines a vision for the government and states the level of priority that needs to be assigned for a particular health domain. On the other hand, programs are referred as a set of interventions with a focused objective for the promotion of health, and prevention, treatment and rehabilitation of health problems. A program usually focuses on a specific health priority and must be adequately designed, budgeted for, monitored and evaluated [6]. We identified documents that addressed (key words) ‘adolescent and young people’, ‘youth’, ‘health’ or ‘mental health and well-being’ in the past 10 years on India’s Official websites of the Ministry of Human Resources Department, Women and Child Development, Youth and Sports affairs, and Family Health and Welfare were searched to extract full text documents of programs and policies. Policy makers or other stakeholders of identified policies and programs were contacted in parallel to obtain their expert views on existing policies and programs in India focusing on adolescent health and mental health. Initially, four national policies were identified: the National Youth Policy (NYP), 2014; National Mental Health Policy (NMHP), 2014; National Mental Healthcare Act (MHA), 2017; and Rashtriya Kishor Swasthya Karyakram (RKSK; National Adolescent Health Program, 2014). Interviews with key informants (see below) led to the identification of two more policies and programs—Sarva Shiksha Abhiyan (SSA), 2014 concerned with education and Yuva Spandana (YS), 2015 concerned with adolescent welfare in the state of Karnataka. Though we identified a range of other adolescent health programs and policies such as the Kishori Shakti Yojna, Balika Samridhi Yojna and the Rajiv Gandhi Scheme for Empowerment of Adolescent Girls by Ministry of Women and Child Development, these policies and programs were not selected because they did not address mental health. Ultimately, six documents were included in the review. Apart from YS, no other regional documents which focused on youth or adolescent well-being were found. Additional sources, including scientific articles and reports pertaining to the selected six policies and programs were identified to retrieve relevant information regarding policy and program development and implementation.

### Key informant interview (KII)

We chose stakeholder analysis as an additional method for policy and program analysis. Stakeholder analysis can be used to understand about relevant actors, their intentions, interrelations, agendas, interests, and the influence or resources they have brought or could bring on decision-making processes during policy development [7]. As a cross-sectional view of an evolving picture, the relevance of stakeholder analysis for predicting any change and managing the same for a policy or a program in the future is time-limited; it should be complemented by other methods of policy analysis [8], which was the rationale for our undertaking a policy review and stakeholder analysis concurrently. We first approached members of the Technical Advisory Group of the RKSK and, using snowball sampling, identified other experts in adolescent health in India. Ultimately, 11 key informants agreed to participate. The interview guide addressed the perceptions of adolescent mental health in India; and views regarding the policies and programs which addressed adolescent mental health concerns, in particular the strengths and gaps in their implementation. All interviews were conducted in English and audio-taped; and later transcribed verbatim. On an average, the interviews lasted for 45 min.

### Data analysis

We applied the ‘policy analysis triangle’ framework [9] to analyze the policy and program documents. This framework recommends analytical attention to four inter-related aspects of a policy, i.e., the context; the content; the actors; and the process. These categories were further operationalized for analysis. For example, the issue of context was explored in terms of the types of mental health problems and their determinants while the content was explored in terms of the strategies and resources to address these problems. The actors were defined in terms of identification of major providers and their roles. The policy process was defined as focusing on participatory decision-making and policy implementation, particularly related to youth involvement.

**Fig. 1:**
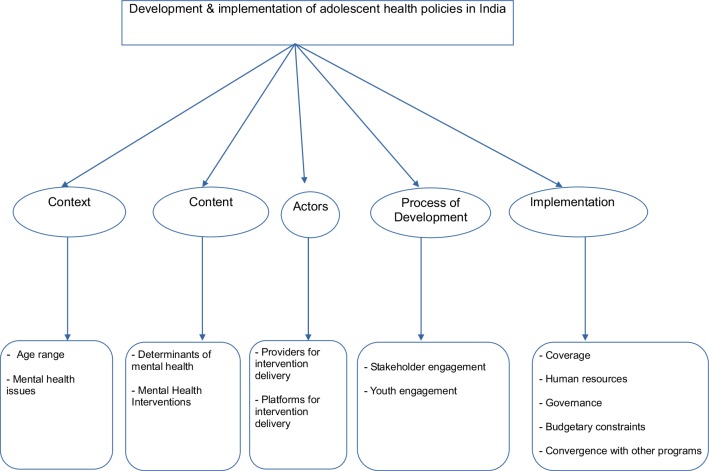
Overview of thematic framework

A five-stage thematic analysis approach was used for analysis of the selected policies and programs and key informant interview transcripts [10]. First, all the policy documents and interview transcripts were read a number of times to ensure adequate immersion in the data and relevant notes were made in each policy and program document and transcript. Upon achieving familiarization with the data, the next stage involved identification of a thematic framework. The themes covered in the policy triangle framework and interviews provided the initial framework, which was refined based on the additional themes and subthemes inductively derived from the data sources. In the third stage, the policy and programs and interview transcripts were indexed/coded. In the fourth stage, data were organized or “charted” according to each theme/sub-theme to include data from different policy documents and key informants. In the final stage of analysis, each chart was examined separately, and a process of mapping and interpretation was undertaken (i.e., established charts were used to explore the range and nature of phenomena and any emerging associations between subthemes were identified in order to explain the findings). Saturation was defined at a point during coding, when two authors (KR and SS) agreed that no new codes were emerging [11]. The final thematic framework is shown in Fig. 1.

## Results

Three national policies, two programs (one national and one regional program), and one national act were selected for the review. The NMHP and the MHA focus on mental health while the SSA has specific focus on universal basic elementary education to children between the ages of 6 and 14 years. The RKSK is the only specific policy related to adolescent health and has the vision that adolescents are able to realize their full potential by making informed and responsible decisions related to their health and well-being. The youth policies (NYP and YS) focus on holistic development of young people. Table 1 presents the summary of each policy and program, its goals, its rationale and process of development. Of the 11 key informants (4 females and 7 males), four were members of the TAG of the RKSK, five were senior researchers in adolescent health in leading institutions and two were members of the leading UN agency working with the Government of India on adolescent health (United Nations Population Fund—UNFPA).

**Table 1:** Summary of the reviewed policies and programs

Policy and program	Description	Need and rationale	Process of development	Goal/s	Youth engagement in policy development
Sarva Shiksha Abhiyan (SSA)—2011	National Elementary Education Program	To provide basic education to children despite their socio-economic backgrounds and physical status, and constitute a sound school environment for their overall development of the child	Consultations with State Education Secretaries, educationists, representatives of teacher unions, voluntary organizations and civil society organizations, led by the Ministry of Education	To achieve universal access to education and retention of students; bridging gender and social class gaps in education; and enhancement of learning levels of children	No specified role of youth in policy development
National Youth Policy (NYP)—2014	National Youth Development Policy	To generate awareness among youth and various stakeholders in India about the overall development of youth and their contribution to nation building	Multiple rounds of consultations across the country were organized, led by the Ministry of Youth Affairs and Sports; no details available about these consultations	To empower youth to achieve their full potential and through them enable India to find its rightful place in the community of nations	No specified role of youth in policy development
National Mental Health Policy (NMHP)—2014	National Mental Health policy	To draft a set of guidelines for mental healthcare in India with focus on disease burden, treatment, management and provision of services	A policy group of experts constituted by the Ministry of Health and Family Welfare, and comprising mental health professionals and persons representing the lived experience and families synthesize evidence and conducted five regional consultations with diverse stakeholders	To decrease the treatment gap, disease burden and extent of disability due to mental illness, taking in to account the Indian sociocultural realities, promoting integrated, evidence-based care and effective provision of quality services	No specified role of youth engagement in policy development
Rashtriya Kishor SwasthyaKaryakram (RKSK)—2014	National Adolescent Health Program	To create a holistic approach for adolescent healthcare with a special focus on nutrition, sexual and reproductive health care, non-communicable diseases, substance misuse, injuries and violence in India	Extensive consultative process with many organizations and experts led by the Ministry of Health and Family Welfare with support from UNFPA	To envisage adolescents in India to be able to realize their full potential by making informed and responsible decisions related to their health and well-being	No specified role of youth engagement in policy development
Yuva Spandana (YS)—2015	State (Karnataka) Youth Program	To ensure that the state government works collaboratively with the recommendations mentioned in the national youth policy of India and implements it for the well-being of youth of the State	Desk review and stakeholder and expert consultations led by the Department of Youth Empowerment and Sports, Government of Karnataka, in collaboration with the Centre for Public Health, NIMHANS	To reach, engage and empower youth of Karnataka to facilitate their overall development	Youth from schools and colleges were invited to share their views on their needs
Mental Healthcare Act (MHA)—2017	National Legislation on Mental health care	To formulate laws and legislation with correspondence to mental health care practices in India and ensure that they are implemented across various sectors, stakeholders, and institutions	Extensive engagement of diverse mental health stakeholders, including mental health professionals, persons with lived experience, families and policy makers, led by the Ministry of Health and Family Welfare	To entitle the rights of people with mental illness to health and social care and a life with dignity, and to ensure that the law is aligned with the UN Convention on the Rights of Persons with Disabilities (UNCRPD)	No specified role of youth engagement in policy development

### The context

Adolescents are referred in a variety of ways in these documents, notably through terms like the young, youth, adolescents and young adults. The RKSK defines the period of adolescence as 10–19 years of age and ‘young people’ as 10–24 years of age; the NYP defines ‘youth’ as 15–29 years of age while the MHA defines ‘minor’ as those persons below the age of 18 years. While key informants strongly recommended the need for recognition of phases of adolescence, notably the marked developmental differences between early and late adolescence, this was not evident in any of the policies or programs.

“We need to have sub-sets for adolescents just like child health has done, neonatal, infant, child, etc. This helps to channelize treatment for each identified sub-set. For an adolescent, there is no specialist as such. It is just the Pediatrician, the pediatricians are also not sure till what age do they check these children, is it 12 or 18 years? Nothing is specified in the policies.” (Senior researcher)

‘Stress’, ‘anxiety’ and ‘depression’ were identified as the ‘common mental health problems or difficulties’ in the reviewed policies/programs and even by the key informants. The policies recognize that addressing suicide attempts and suicidal tendencies among adolescents is crucial (NYP) and that bullying (including cyber bullying) seems to be increasingly linked with adolescent suicide (RKSK). These policies also recognize that use of tobacco (cigarette/*bidis* and chewing tobacco and tobacco products), alcohol, and other intoxicants is common among the young people in both rural and urban areas (RKSK, NYP). Learning difficulties are acknowledged only in SSA. There is no mention of disruptive behavior disorders. The documents explicitly recognize that adolescent well-being is strongly affected by social and educational determinants operating at the individual, family and community levels, for example bullying, safe and supportive schools. Other social determinants such as social norms and parental conflict are less acknowledged.

“Normal pressure like daily stress, peer pressure, parental pressure, academic pressure, etc., creates mental health issues for adolescents. Depression, anxiety, aggression and suicidal tendencies are major mental health concerns among adolescents.” (Senior researcher)

### The content and actors

We broadly classified adolescent mental health interventions as either preventive or treatment. Table 2 provides an overview of the mental health interventions and their recommended delivery agents. Most interventions were intended for delivery on the community platform, notably preventive interventions such as substance use awareness generation camps and group meetings at teen clubs. These interventions were generally delivered through non-specialist providers including youth volunteers, Peer Educators (PE), Accredited Social Health Activists (ASHA) and Auxiliary Nurse Midwives (ANM). Youth engagement is mentioned in two documents. The RKSK mentions the role of the PE serving to sensitize adolescents on health issues and facilitating referral to health services. A mobile app (‘Saathiya Salah’) has been designed to guide PE with key information on adolescent health. The YS program proposes two cadres of providers, i.e. ‘Yuva Parivarthakas’; YP—meaning change agents of youth; and ‘Yuva Mithras’; YM—meaning friends of youth. They serve as psychosocial support service providers in the state (Karnataka). The only mental health intervention at the family level was guidance sessions regarding adolescent health concerns provided by YM and YP (YS program).

**Table 2:** Mental health interventions for adolescents in India: recommendations of the reviewed policies and programs

Platforms	Content	Delivery agents
Community	Preventive 1. Educating adolescents about mental health concerns through Sabla^a^ and NYKS^b^ teen clubs—(RKSK, NYP) 2. Peer Education (PE) program to build awareness about mental health and other adolescent health concerns—(RKSK) 3. Awareness generation on ill—effects of drug/substance abuse (NYP)	ANM, ASHA, Peer educators, youth volunteers—(RKSK, NYP)
	Treatment	
	1. Conducting individual and group support/guidance sessions for youth and their families—(YS)	Yuvasamalochaka and Yuvaparivarthakas—(YS)
Digital	Preventive 1. ‘Saathiya Salah’ mobile app acts as cost-effective information platform for the adolescents (RKSK)	–
	Treatment 1. Providing telephonic guidance (Individual and group) for youth and their families—(YS) 2. Saathiya Helpline (in the app) as an e-counselor (RKSK)	–
Family	Preventive 1. Conducting group/individual sessions with parents to sensitize them on adolescent health needs—(RKSK)	Medical Officer or ANM—(RKSK)
School	Preventive 1. Life skills education—(NMHP; SSA; and RKSK) 2. Educating adolescents about mental health concerns through classroom teaching as per curriculum—(RKSK)	Teachers and skilled trainers—(NMHP; SSA; and RKSK)
	Treatment 1. Screening children for mental health problems and other health problems (RKSK)	Doctors, ANM, pharmacist—(RKSK)
Healthcare facility	Treatment 1. Providing counselling services at AFHC and providing referrals to specialist whenever required—(YS and RKSK)	ANM, ASHA, dedicated counsellor, Yuvasamalochaka and Yuvaparivarthakas (YS), and Medical officer—(RKSK)
	2. Treating new and referral cases and emergency cases at district and specialist hospitals	Psychiatrist, Pediatrician—(RKSK, NMHP)

^a^Sabla is a centrally sponsored program of Government of India which was initiated to enable adolescent girls for self-development and empowerment by provision of various facilities like health checkup and referral services, nutrition and health education, counseling/guidance on family welfare, life skill education

^b^Nehru Yuva Kendra Sangathan (NYKS) is an autonomous organization of the Ministry of Youth Affairs and Sports, India. It is the largest grass root level apolitical organization in the world, catering to the needs of more than 8 million non-student rural youth enrolled through 2.95 lakh village based youth organizations called Youth Clubs in the areas covering education and training, awareness generation, skill development and self-employment, enterprise creation, thrift and cooperation, besides development of the body through sports and adventure and mind through sustained exposure to new ideas and development strategies

At the school platform, life skill education through class room curricula was a key preventive intervention mentioned in NMHP and SSA. Teachers or skilled trainers were the delivery agents. Signs and symptoms of many mental disorders first appear during adolescence and thus school teachers must be well trained in mental health promotion and pay individual attention to them (NMHP). Treatment interventions dominated at the healthcare facility platform and were delivered at various settings, i.e., at Adolescent Friendly Health Clinics (AFHC), Primary Health Centre (PHC), Community Health Centre (CHC) and district hospitals. These interventions were delivered by specialists and trained professionals like doctors, counselors and nurses. The focus was to provide adequate guidance and care to adolescents who approach directly or were referred to these facilities.

### Process of policy and program development

These policies and programs were developed through multiple rounds of consultations with experts from government as well non-government organizations including multiple stakeholders such as law makers, health, mental health professionals and teachers. However, details on the selection of the experts, number of rounds of consultation that had taken place, and duration and nature of these consultations were lacking. The role of youth engagement in process of development was not clearly addressed in any of the policy/program.

### Challenges to implementation of policy and programs

This theme was mainly addressed through the key informant interviews. The key informants expressed concern about the patchy implementation of the policies and programs, as reflected in the lack of details on the scope and delivery of the proposed mental health interventions.

“There is a lack of clarity on mental health as a public health issue. There is not enough information available on the issues faced by the adolescents. Definition of the problems are very important and then the policies should be written. For adolescents there is no such information available. Pediatricians are still the main doctors for adolescents. Dermatologists have specific interest. Gynecologists are there for other problems but not for mental health or nutrition…there is lack of awareness on and absence of specialized care for adolescent mental health issues.” (Member of funding agency)

However, state level variations were also described, for example the better delivery of the PE component of the RKSK in Madhya Pradesh and West Bengal was acknowledged as this component was delivered through local NGOs in these states. In Meghalaya, Kerala and Karnataka, the functioning of AFHCs as expected was attributed to availability of infrastructure, political willingness and dedicated human resource.

“Meghalaya has a very interesting model of adolescent friendly health clinic… the staff in the clinics are very dedicated, the privacy is maintained, and confidentiality is assured to adolescent clients. These clinics are, a place where one will feel like going in. They have tried to convert it into a non-health setting environment…In West Bengal the system is in place, they have 420 counselors who were trained by the NGOs like CINI and Center for Catalyzing Change.” (Member of the TAG of the RKSK)

Common reasons mentioned for poor implementation were the fragmented governance and lack of inter-sectoral collaboration, budget constraints, and scanty human resources. A few states such as Delhi, Gujarat, Karnataka and Kerala had taken initiatives to integrate mental health into general health care. However, the lack of inter-sectoral collaboration and convergence were the major challenges, with various interventions falling into the domains of diverse departments and ministries such as education, health, women and child development and youth development, with little effort to coordinate their activities. Concerns were also expressed about the hierarchical structure of personnel at center and state level.

The concerns were mainly related to inter-personal challenges of working in the central and state government. Central government officials would have meetings with minimal involvement of state government officials and later forward their recommendations for implementation at the state level. The state government officials felt left out during these decision-making activities, which further stalled the implementation process. If these activities are well managed and streamlined, then this could lead to a more mutually respectful and effective working environment at state and central government levels.

“No particular ministry is involved, it is usually the health ministry and health department which is involved in the mental health policy development. All ministries have different mandate; the basic tendency of all personnel is of achieving targets made for the ministry. All the senior managers are too focused on their targets. Then there are ego hassles amongst senior authorities.” (Member of funding agency)

The fragmentation of governance between center and states also affected budgetary allocations which were, in any event, relatively modest for the proposed goals of the documents. The central government framed guidelines and designed interventions without any consultation with state government officials. Unaware of these central government directives, the state governments do not seek funds from the central government or make adequate allocations from their own budgets for implementing adolescent mental health initiatives.

“There is no clear communication between center and states about what policy is developed or how will the funds be used for its implementation. Every year the center declares new initiatives for adolescents but then the state is unaware of how to implement them.” (Member of funding agency)

Two major human resource challenges were mentioned. The first was regarding front-line providers, and the challenges were the restricted salary structure which were either set too low to retain these providers. The second challenge was regarding the lack of clearly defined procedures for the recruitment and training of new providers specifically recruited for adolescent health (under the RKSK).

“Salary structure for adolescent health service providers remains always a question. Offering them Rs. 12000–15000 is not what they would like to work on. More remuneration is required.” (Member of funding agency)

“RKSK guidelines had mentioned two counsellors to be placed at the facilities but nothing more was explained about it in the guidelines, such as the eligibility criteria, qualification of the counsellors, experience, etc. Until it is well defined how can someone implement or work on that? We need very highly qualified counsellors to respond to the issues of adolescents”. (Member of the TAG of the RKSK).

These human resource challenges were compounded by the lack of trained mental health professionals and unclear policy guidelines for their recruitment and sustainability.

“There is dearth of mental health professionals in this country… existing mental health policy people is scanty, 2 or 3 people are expected to run a mental health policy for 1300 million people, even these people lack sound training in mental health, then how can we expect to make them run a successful MH policy or program. There is an issue of institutional capacity in terms of quality and quantity… we need a special technical resource group for mental health to operationalize the same.” (Senior researcher)

Across all these themes, a recurrent cross-cutting finding was the lack of clear communication within and across various ministries and departments.

“Human resources for implementing mental health programs in our country has been a consistent issue, the next being the ruling political party and its communication within and across various departments. For a moment let’s say even though there is a strong political will to implement a program, the next big issues arise, where is the money to recruit resources for implementation?” (Senior researcher)

## Discussion

This paper describes the approach taken in current policies and programs in India to address the mental health of adolescents, and the challenges in implementing these policies and programs. We employed the health policy analysis framework, widely used in other areas of health [9], to analyze 6 policies and programs and concurrently interviewed 11 key informants to identify challenges in the implementation of the documents. As in the wider field of adolescent and youth health, there is no conformity of the age ranges addressed by these documents, with some adopting the orthodox WHO definition of adolescence, while others extending the age range as recommended as being more developmental wise accurate [12]. While mental health is acknowledged in health, education and adolescent health documents, and adolescent mental health in mental health documents, there is inadequate depth (for example, in addressing the full range of mental health problems) and detail (for example, on addressing the major barriers in implementing the proposed interventions).

Stress, anxiety and depression were the most frequently identified mental health problems. On the other hand, psychoses, substance use disorders and self-harm, which are less common, but often much more serious and enduring, are poorly addressed [13]. Similarly, while some of the social determinants of mental health are discussed, for example school attendance, attainment and supportive school environments, others related to families, communities, social norms and childhood experiences are most omitted. There is also little acknowledgement of principles of equity and social justice, for example in the different needs of adolescents in rural regions, those in vulnerable or marginalized contexts (such as homeless or sexual minorities), those living with disabilities and those who experienced neglect and abuse in childhood. The bulk of interventions were preventive in nature, with a dominance of school-based curricula, and most had little details on implementation or the rationale for their selection. Interventions targeting families or provision of psychosocial interventions for adolescent with mental disorders were poorly described, even in the specific documents pertaining to mental health or adolescent health. While task-sharing with front-line providers (such as ASHA workers) was a frequent recommendation, the training, supervision and financing models to enable this role, or their coordination with other platforms of delivery for addressing more intensive interventions, were largely ignored. The lack of youth engagement in the development of the documents and their limited role, in the form of peer educators, in the delivery of interventions, or governance, is another major finding. Technology enabled interventions are few, and include the Saathiya salah app to enable peer educators with information to discuss health concerns and provide appropriate guidance, referral and telephone counselling.

Apart for the limitations of the existing policies and programs, the lack of alignment across them and the fragmentation of governance of adolescent mental health between ministries and departments, are likely to pose major barriers to their effective and efficient implementation. Despite the plethora of policy and programs, there are severe budgetary and human resource constraints which greatly limit the deployment of the proposed interventions. Many of these challenges in the implementation of policy and programs, such as lack of inter-sectoral collaboration and role of governance, budgetary constraints and scanty human resources, are similar to those reported in the context of other health policies [14, 15]. At the heart of these challenges is the weak technical capacity, poor motivation, inefficient management practices, and fragmentation of governance of health. This results in lack of clarity of mandates and roles, particularly for population and community-based interventions, and consequent gaps in delivery. Within the decentralized health governance framework in India, coordination is both horizontal (between ministries and departments) and vertical (from center to state to district). Addressing these systemic challenges, as envisaged in the National Health Mission, will be an essential structural strategy to address adolescent mental health. Human resource shortages could be addressed through community health workers, lay counsellors and peers, all of whom are numerous and relatively lower cost than professional health care providers. The greater success that some states have had in implementing programs with such providers through NGOs offers an example of how governments can partner with civil society.

Going forwards, policies and programs need to be better aligned with evidence-based practices emerging from both scientific studies and program implementation experience. While this evidence, often generated by NGOs working in partnership with governments or academic institutions, provide robust support for the acceptability and effectiveness of interventions delivered in community and school settings by non-specialist providers, policies and programs need to systematically address the barriers to the scaling up of such findings. This requires, for example, sustainable strategies for training, supervision and monitoring the roll-out of such interventions which, in turn requires the active engagement of other sectors, including skills building initiatives such as distance learning programs, capacity building by health care providers from diverse disciplines such as mental health professionals and pediatricians, and the mobilization of youth leaders and change agents.

The limitation of this study was the possible exclusion of some key documents and informants on account of the snowball sampling procedure. However, the rapid saturation of our interview findings suggests we were unlikely to have missed a key theme in our results. While there have been important achievements in national health indicators in India, e.g., on child mortality, and on social development indicators, e.g., delayed age of marriage, which will have beneficial impacts on adolescent health, there is also a need to simultaneously address the specific determinants of adolescent ill-health and their consequences. We recommend a broader, life-course approach, to addressing these needs, with interventions starting from childhood and extending into young adulthood, the active engagement of young people in the design and delivery of the interventions, and the coordinated involvement of diverse sectors and platforms for prevention and treatment. Equity must be a guiding principle throughout: one size does not fit all and contextual realities and specific sub-group vulnerabilities should be considered while framing and implementing policies. Interventions should be prioritized based on their evidence, scalability and equity. While it is too early to recommend specific digital interventions, the rapid growth of this technology and their embrace by adolescents clearly indicates the need to develop and evaluate their utility in promotion and prevention (e.g., through information provision and counselling), but also to regulate those elements which are potentially harmful. Treatment interventions should ideally be integrated in routine health care and made easily accessible, for example through school-based delivery of counselling interventions.

The national adolescent health policy (the RKSK) is potentially the most appropriate policy for such a coordinated and integrated approach, but must work closely with the education and mental health policies to ensure efficiency and alignment of all proposed interventions, from pooling of financial resources to selection of interventions and human resources management for delivery of interventions. India is home to the largest number of adolescents and young people in the world and a coordinated effort to correct the deficiencies in the existing policies and to coordinate their implementation to optimize coverage and impact will have national and global impacts on addressing the burden of mental and substance use disorders in adolescents.
